# A National County-Level Assessment of U.S. Nursing Facility Characteristics Associated with Long-Term Exposure to Traffic Pollution in Older Adults

**DOI:** 10.3390/ijerph15030487

**Published:** 2018-03-10

**Authors:** Yi Wang, Hao Fan, Aniruddha Banerjee, Anne M. Weaver, Michael Weiner

**Affiliations:** 1 Department of Environmental Health Science, Indiana University Fairbanks, School of Public Health, Indianapolis, IN 46202, USA; fanhao@umail.iu.edu (H.F.); weaveran@iu.edu (A.M.W.); 2 Department of Geography, School of Liberal Arts, Indiana University-Purdue University in Indianapolis, Indianapolis, IN 46202, USA; rbanerje@iupui.edu; 3 Department of Medicine, Indiana University School of Medicine, Indianapolis, IN 46202, USA; mweiner@iu.edu; 4 Regenstrief Institute, Inc., Indianapolis, IN 46202, USA; 5 Center for Health Information and Communication, U.S. Department of Veterans Affairs, Veterans Health Administration, Health Services Research and Development Service CIN 13-416, Richard L. Roudebush VA Medical Center, Indianapolis, IN 46202, USA

**Keywords:** traffic pollution, nursing home, indoor air, minority, occupancy, profit

## Abstract

Long-term exposure to ambient air pollution increases disease risk in older adults. Nursing facilities located near major roadways potentially expose older adults to traffic pollution. No studies, however, have described the association between nursing facilities and traffic pollution. We obtained data on facility- and census-tract-level characteristics of 15,706 U.S. facilities from the Medicare Nursing Home Compare datasets. We calculated distance to major roadways and traffic density for each facility. In the contiguous U.S. (as of 2014), 345,792 older adults, about 27% of residents in non-hospital facilities, lived within 150 m major roadways (A1 or A2) in 3876 (28% of sampled) facilities. Nationally, for-profit facilities, high-occupancy facilities, and facilities in census tracts with higher percentages of minorities were more likely to have higher exposure to traffic. Counties in Virginia, New York City, and Rhode Island have the highest percent of residents and facilities near major roads. Nationally, over one-quarter of sampled facilities are located near major roadways. Attributes potentially associated with higher exposure to traffic included “for-profit” and “higher minority census tract”. Proximity to major roadways may be an important factor to consider in siting nursing facilities. Our results inform potential intervention strategy at both county and facility level.

## 1. Introduction

Long-term exposure to ambient air pollution, particularly traffic-related pollution, has been shown to increase the risk of a wide range of adverse health outcomes in older adults. These adverse outcomes include cardiovascular diseases [1,2,3], cognitive decline [4,5], stroke [6], depressive symptoms [7,8], Alzheimer’s disease [9,10], and diabetes mellitus [11]. A number of epidemiological studies have shown living in proximity to major roadways, a marker of long-term exposure to traffic-related pollution, increases the risk of hypertension [3,12], cognitive decline [13], and atherosclerosis [14,15]. Increased risk of various health outcomes can lead to higher healthcare cost and lower quality of life.

Nursing-home residents—who often select long-term care facilities where needed services are available to them, rather than where they would otherwise choose to reside—are vulnerable to ambient air pollution near their residences. More than 1.4 million residents were living in US nursing homes in 2014, corresponding to 2.6% of the population over 65 years of age, and 9.5% of the population over 85 [16]. Residents of these facilities spend most their time indoor and yet there is lack of specific regulations beyond quality of care standards to ensure air quality in nursing facilities, except for state-specific requirements, such as keeping ducts and air handling equipment clean, and cleaning carpet every month, etc.

Published studies, however, have not described the possible association between nursing facilities in the U.S. and the long-term exposure to traffic pollution that many of the residents may experience. Traffic pollution is a major contributor to ambient air pollution and a main predictor for intra-urban variations. Given the prevalence of older adults in nursing facilities, and the residents’ vulnerability to pollution from traffic, the objective of this study was to characterize and identify attributes of U.S. nursing facilities that are potentially associated with higher exposure to traffic pollution, as indicated by proximity to major roadways and traffic counts, widely used markers of long-term exposure to traffic-related pollution.

Americans spend an average of 68% of their time at home [17], and the time spent indoors by older adults in need of care is likely much higher. An individual’s total exposure to PM2.5 represents the sum of his or her exposure to particles of ambient or outdoor origin (found outdoors or indoors) and particles of indoor origin. Two arguments support the use of ambient measures as a proxy of total exposure in epidemiologic studies. First, studies show that ambient level of pollutants such as PM2.5 is a relatively good surrogate of personal exposure to PM2.5 of ambient origin [18,19,20]. Second, studies show that levels of PM2.5 of ambient origin are uncorrelated with levels of PM2.5 from indoor sources [19,20].

We graphed variations by U.S. county in the percent of nursing residents and facilities potentially with higher exposure to traffic pollution. We identified the top 1, 5, and 10% U.S. counties with the highest percentage of nursing residents and facilities near major roadways. We hypothesized that some facility characteristics and socio-economic-related characteristics of census tract where facility is located would be associated with higher exposure to traffic pollution.

## 2. Methods

### 2.1. Facility-Level and Census-Tract-Level Characteristics of Nursing Facilities

We obtained nursing-home provider data from the Nursing Home Compare datasets [21]. The provider datasets used in this analysis include street address, facility-level characteristics of every Medicare- and Medicaid-certified nursing home in the U.S. as of 1 April 2014. We also derived census-tract-level characteristics of a facility based on the census tract where a facility is located.

### 2.2. Facility-Level Characteristics

We included facility-level characteristics such as ownership (for profit, government, nonprofit), certification (Medicare but not Medicaid; Medicaid but not Medicare; or both Medicare and Medicaid), council type (resident, family, or both), capacity (number of federally certified beds), and occupancy (number of occupied beds).

### 2.3. Census Tract-Level Characteristics

We used characteristics of census tracts (2010 U.S. Census) in which nursing facilities are located as a proxy of characteristics of the facilities at census tract level. These characteristics include percentage of urban area (urban area divided by total area, for each tract), percentage of non-white, and median household income. In all analyses, we dichotomize these tract-level characteristics by respective median values to obtain percent of urban areas (high versus low), percent of nonwhite (high versus low), and median household income (high versus low).

We use U.S. Census designation of U.S. regions to group 48 states in contiguous U.S. (excluding Alaska, Hawaii, and Puerto Rico) into four regions, including West (states west of Montana Wyoming, Colorado, and New Mexico, inclusive), Midwest (states north of Kansas, Missouri, Illinois, Indiana, and Ohio, inclusive), South (states south of Oklahoma, Arkansas, Kentucky, West Virginia, Maryland, and Delaware, inclusive) and Northeast (states east of Pennsylvania and New Jersey, inclusive).

### 2.4. Additional Facility-Level Characteristics

For completeness of our analysis, we additionally explore care-quality measures of the facilities even though there might not be a direct link between care-quality measures and long-term exposure to traffic pollution. The care-quality measures included overall quality rating derived from rating of three domains, including individual quality rating for health inspection, individual quality rating for nursing home staffing levels (registered nurse + licensed practical nurse + nurse aide), and individual rating for quality measures (function and health status indicators based on Minimum Data Set (MDS) and Medicare claims data) [22]. We focuses on overall ratings of quality of care and more details are included in the Supplementary Materials.

### 2.5. Measures of Exposure to Traffic-Related Pollution

Given that traffic pollution is a major contributor to ambient air pollution and a main predictor for intra-urban variations, we used proximity to major roadways and traffic count, widely used markers of traffic pollution, as the measure of exposure to traffic pollution for older adults living in nursing facilities.

We defined major roadways as A1 (primary roads with limited access or interstate highway) or A2 roads (primary road without limited access) based on Census Feature Class Codes (CFCC) definition. First, we used distance to major roadways as the primary indicator of long-term exposure to traffic-related pollution. Therefore, a facility is consider near major roadways if it is located within 150 m of A1 or A2 roads. Second, we calculated length of A1 or A2 roads within a radius of 300-m circular buffer from each facility, to indicate traffic density. This is our secondary indicator of long-term exposure to traffic pollution. Third, we calculated annual average daily traffic (AADT) counts separately for AADT for total traffic and AADT for multi-trailer combination truck within a 300-m circular radius of each facility as additional indicators of traffic density. Briefly, we obtained 2014 AADT count data for AADT for total traffic and AADT for multi-trailer combination truck from the US Department of Transportation Office of Highway Policy Information [23]. To assign the nationwide AADT count data to each CFCC-defined road segment, we used TransCAD^TM^ 7.0 (Caliper Corporation, Newton, MA, USA) transportation GIS software to overlay AADT data layer with U.S. census TIGER/Line files (2014), so each road segment with CFCC is assigned a value of AADT. We calculated the average AADT based on weight of segments. Because about 59% of facilities did not have A1 or A2 roads within 300 m, the sample size of facilities with data on traffic density (total AADT or combo truck AADT) was smaller (*n* = 6034) than that (*n* = 14,780) for traffic density (length).

### 2.6. Statistical Analysis

We only included nursing facilities that are not located in hospitals. In the analysis involving proximity to roadways, we excluded facilities for which a value of zero was recorded for proximity due to street-level rather than roof-top geocoding on these addresses. We used 150 m as the cutoff to dichotomize distance to roadways, and 300 m as the buffer radius, because the concentration of particulate matter from highway traffic pollution decreases by 50% at 150 m and fades to background level after 300 m [24].

First, we described facility-level and census-tract-level characteristics by distance to roadways (≤ or >150 m), traffic density (length) within 300-m-radius buffer (0 or >0 m) and traffic density (total AADT, combination truck or semi-truck AADT) using the median as cutoff. Second, we estimated prevalence ratios and 95% confidence intervals (CI) to assess the association of facility-level and census-tract-level characteristics separately, with proximity to major roadways, and traffic density (length, total AADT, and combo truck AADT) within 300 m of each facility. Third, to examine regional variations among subgroups of facility-level and tract-level characteristics, we stratified these associations by region (West, Midwest, South, Northeast). Finally, we repeated the first, second and third analyses described above for measures of quality of care and all results related to measures of quality of care were included in the Supplementary Materials.

### 2.7. Normalized County-Specific Percent of Nursing Residents and Facilities near Major Roadways

For each U.S. county, we calculated county-specific percentages of nursing residents living near major roadways among the total residents in nursing facilities. It is important to note that this percent will increase with the county size. As there are more roads, namely, higher total length of A1 or A2 roads, nursing residents are more likely to be located near major roadways. Therefore, we normalized this percent to area and total length of major roads in each county. We included both area and total length because counties with small area could have high length of roads and vice versa.

We repeated this process to derive the percentages of nursing facilities near major roadways among the total number of nursing facilities in each U.S. county, normalized to county area and total length of major roads.

We then assigned county-specific percentile ranks for the two normalized percent measures by ranking the values of all U.S. counties within respective percent measures. We graphed the two normalized percentile ranking measures to show county variations across the U.S. and counties with no residents or facilities near major roads were shown excluded in percentile ranking and shown as blank in our graphs.

## 3. Results

In the contiguous U.S., there were 15,706 nursing facilities, 5.5% (*n* = 869) of which were located in a hospital. These in-hospital facilities were not included in our analysis, as described above, resulting in a sample size of 14,837 nursing facilities in the contiguous U.S.

**Figure 1:**
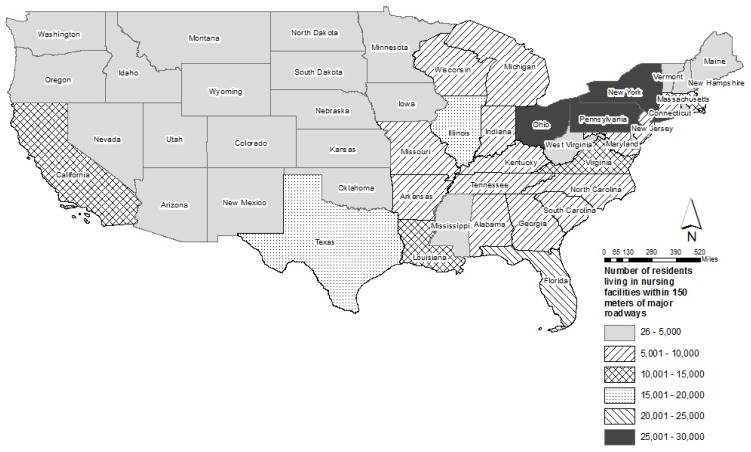
Number of residents living in nursing facilities located near (within 150 m of) major roadways (A1 or A2) in the 48 contiguous U.S. states.

**Figure 2:**
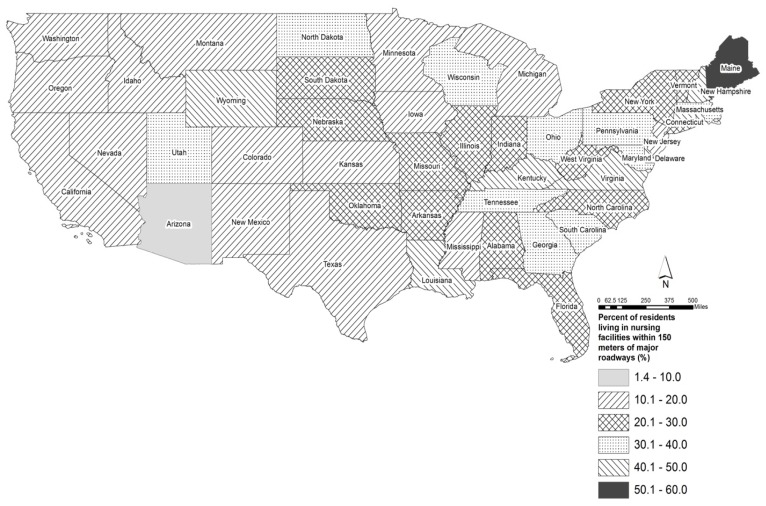
Percent of residents living in nursing facilities located near (within 150 m of) major roadways (A1 or A2) in the 48 contiguous U.S. states.

There were 345,792 older adults living in nursing facilities located near major roadways (within 150 m of A1 or A2); this number is about 27% of the total older adults living in non-hospital nursing facilities. The number of older adults in nursing facilities in each state or district ranged from 26 in Washington, D.C. to 26,945 in Pennsylvania, with a mean of 13,832, a median of 4866 and a standard deviation of 7022. The states with the highest number (more than 20,000) of older adults living near major roadways were Pennsylvania (26.945), Ohio (25,894), New York (25,884), and Florida (21,660) (Figure 1). Among the states and District of Columbia, the median value of percentage of older adults living in nursing facilities near major roadways was 27% (mean value = 25%), with a standard deviation of 12% and ranging from 1% in Washington, DC to 54% in Maine. The states with the highest percentage (more than 45%) of older adults living near major roadways were Maine (54%), New Hampshire (50%), Louisiana (49%), Rhode Island (46%), and Delaware (46%) (Figure 2).

After excluding facilities having a value of zero for distance to roadways (*n* = 57) or missing for distance to roadways (*n* = 937), our sample size in the analysis of distance to roadways was 13,843, 28% of which are located within 150 m of A1 or A2 (Table 1). After excluding facilities with 57 missing values in traffic density (length), our sample size in the analysis of traffic density (length) was 14,780, 59% (*n* = 8746) of which had no A1 or A2 roads within 300 m from facilities, and 41% (*n* = 6034) of which had at least one or more segments of A1 or A2 roads within 300 m from facilities (Table 1). We presented for traffic density (total AADT and combo truck traffic) in Supplementary Materials Table S1.

### 3.1. Facility-Level and Census-Tract-Level Characteristics

Compared to nonprofit facilities, for-profit facilities were more likely to have higher level of traffic pollution. For example, for-profit facilities were 9.6% more likely to be located near major roadways (Table 2) and among facilities that has A1 or A2 roads within 300 m buffer, 37.3% are more likely to be exposure to higher total traffic (Table 3). Facilities that had councils comprised of residents or both residents and family were more likely to be located near major roadways than those with no council (Table 2). The results were largely similar for total traffic and truck traffic even though they are not statistically significant (Table 3). Among facilities with A1 or A2 roads within 300 m buffer, higher-occupancy facilities were more likely to have higher exposure to total traffic and truck traffic (Table 3) although the results for proximity to major roads is the opposite and not statistically significant (Table 2). The results for certification type are conflicting between exposure measures using traffic count and proximity to major roads (Table 2 and Table 3).

Among facilities with A1 or A2 roads within 300 m buffer, compared to tracts that have higher percent of whites, tracts with higher percent of minority are more than twice as likely to have higher exposure to total traffic and truck traffic, but results for proximity to roads is the opposite. These results are statistically significant across all exposure indicators (Table 2 and Table 3). The results for urban coverage and household income vary across exposure indicators.

We presented results of quality of care associated with traffic density (AADT) mostly in Supplementary Materials Tables S2–S4. Overall, nursing facilities with higher overall rating of quality of care were at least 11% less likely to be located near major roadways (Supplementary Materials Table S3). This association also held in the analysis of traffic density (length) and in the analysis of traffic density based on AADT combination truck but not total AADT (Supplementary Materials Table S4).

### 3.2. Variation by Geographic Region and by U.S. County

We observed variations by geographic region in the association between proximity to major roadways and facility-level and tract-level characteristics. The associations hold across U.S. regions for occupancy, percent of urban area, and percent of non-whites but not household income. For facility-level characteristics such as ownership, certification, and council type, the associations for facilities in the West are the opposite of those for the entire U.S. (Supplementary Materials Table S5). The results in the analysis of traffic density (length) were generally similar to those in the analysis of proximity to major roadways (Supplementary Materials Table S6). Regarding quality of care measures such as overall rating, the associations hold for the Midwest and Northeast but not the West and South (Supplementary Materials Tables S7 and S8).

**Figure 3:**
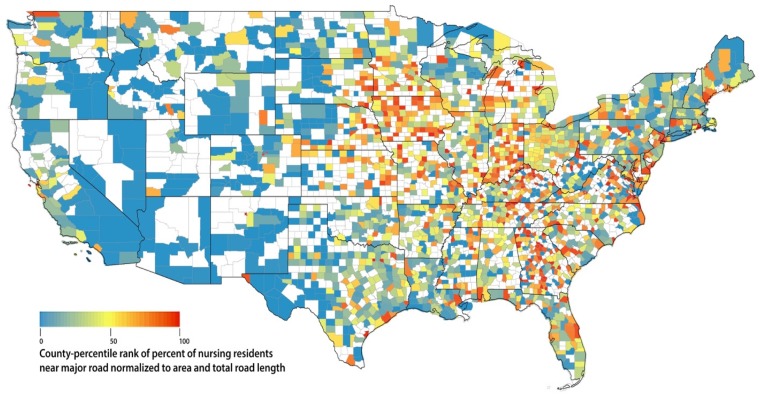
Percentile rank of percent of nursing residents near major road, normalized to county area and total length of major roadways within each county. Blank indicates no nursing resident near major road in that county.

**Figure 4:**
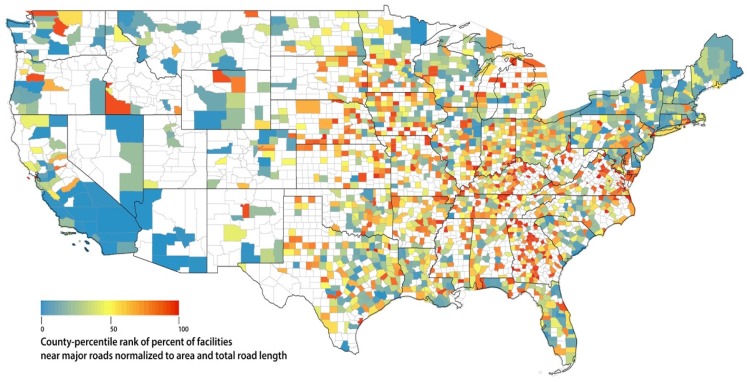
Percentile rank of percent of nursing facilities near major road, normalized to county area and total length of major roadways within each county. Blank indicates no nursing facility near major road in that county.

We plotted the respective percentile rank of percent of nursing residents near major roadways and percent of facilities near major roadways, respectively in Figure 3 and Figure 4. We also included a list of counties with top 1, 5, and 10% of the respective percentile ranking, in Supplementary Materials Tables S9 and S10, in accordance to Figure 3 and Figure 4. Not surprisingly, there are significant overlap between the two lists. Top 1% counties on both lists include many counties in Virginia around Washington DC, many counties in and around the New York City and Bristol County in Rhode Island. Those with highest percent (top 1%) of facilities near major roads but not highest (top 1%) residents near major roads are Los Alamos County in New Mexico, Trousdale County, and Moore County in Tennessee, and Union County in Indiana. The majority of top 10% counties on both lists are located east of Mississippi River.

## 4. Discussion

This is the first published study that discusses potential long-term exposure to air pollution particularly from traffic in older adults living in nursing facilities, associated with proximity to major roadways, and it is the first study to identify facility-level and census tract-level nursing facility characteristics that may be associated with higher exposure to air pollution from traffic.

We found that a large number of nursing home residents are potentially exposure to elevated levels of air pollution from traffic. As of 2014 in the contiguous U.S. states, 345,792 older adults, about 27% of the total population in non-hospital nursing facilities, lived in 3876 nursing facilities located near major roadways (A1 or A2). We observed variations among 48 U.S. states in proportion and total number of nursing home residents living near major roadways. At 26,945 people, Pennsylvania had the highest number of nursing residents living near major roadways while the states with highest percentage of residents living near major roadways were mostly in the Northeast, including Maine (54%) and New Hampshire (50%).

On a county basis when normalized to county area and total length of major roads, the highest percent of nursing residents near major roads include many counties in Virginia around Washington DC, many counties in and around New York City and Bristol County in Rhode Island. The highest normalized percent of nursing facilities near major roads additionally include Los Alamos County in New Mexico, Trousdale County and Moore County in Tennessee, and Union County in Indiana, in addition to those counties around New York City and DC metro and Bristol County described above. The majority of top 10% counties are located east of Mississippi River.

We also identified attributes of nursing facilities that are potentially associated with higher exposure to traffic pollution. We found that for-profit facilities are more likely to be located near major roadways and have higher traffic exposure. Higher-occupancy facilities were more likely to have higher exposure to traffic. Facilities located in census tracts with higher percent of minority are more likely to have higher exposure to traffic. The relationship of proximity-based and traffic-based exposure indicators with characteristics needs to be explored further for council composition, certification type, urbanity and median household income of the census tract. The results may be explained because for-profit developers are likely site their facilities in locations with easy access to major roadways. It is not surprising to observe a statistically significant correlation between percent of minority and exposure to traffic pollution. As additionally presented in Supplemental Materials, nursing facilities with higher overall care quality ratings are less likely to be located near major roadways.

Previous literature has shown a positive association between indoor air pollution and risk of adverse respiratory health in older adults living in nursing homes. A large epidemiological study of 600 older adults from 50 nursing homes in seven European countries reported that indoor PM increased risk of wheezing, breathlessness, and even chronic obstructive pulmonary disease in older adults [25]. It highlighted the importance of air quality for health in older adults living in nursing facilities even at a low level. This is particularly important given that nursing facilities may be under intense scrutiny for meeting quality-of-care standards, but the regulations on air quality are neither specific nor well-defined. Air pollution is a modifiable risk factor for diseases prevalent in older adults. Proximity to major roadways may be an important factor to consider in siting nursing facilities.

Our studies have potential limitations. We used distance of each nursing facility to major roadways and AADT traffic counts as indicators of long-term exposure to traffic pollution. We recognized that actual exposure may depend on ventilation and filtration systems of each facility, and the residents’ individual activities. Nonetheless, policymakers frequently use distance to major roadways as a criterion for siting buildings such as schools, largely because it is easy to measure and understand.

On the other hand, the study featured a large, nationwide dataset of nursing facilities with detailed data about facility characteristics and characteristics of census tracts where facilities are located. This is the first published study that described the relationship between long-term exposure to traffic pollution and nursing facility characteristics. The results of the current study could facilitate research and conversation on how nursing facility siting, and facility characteristics affect health outcomes in older adults living in nursing facilities.

Additionally, we explored county variation in percent of nursing residents and percent of facilities near major roadways with the aim to inform potential intervention strategy at both county and facility level. Our percentile ranking of percent of nursing residents near major roadways has potential public health implication while our percentile ranking of percent of facility near major roadways has potential policy implication.

## 5. Conclusions

In the contiguous U.S. as of 2014, about 27% of the population in non-hospital nursing facilities live near major roadways, potentially leading to increased exposure to elevated levels of air pollution from traffic. We identified attributes of nursing facilities that are potentially associated with higher exposure to traffic pollution, including for profit, higher-occupancy, and location in a census tract with higher percent of minority. The results may vary by U.S. region. Nationally, Pennsylvania has the highest number of nursing residents living near major roadways, while two of the top three states with the highest percentage of residents living near major roadways are in the Northeast (Maine, 54%, and New Hampshire, 50%). Proximity to major roadways may be an important factor to consider in siting nursing facilities. On a county level, many counties in Virginia around Washington DC, many counties in and around New York City and Bristol County in Rhode Island have the highest percent of nursing residents and facilities near major roadways, normalized to county area and total road length. Our results inform potential intervention strategy at both county and facility level and thus have potential public health and policy significance.

## Figures and Tables

**Table 1:** Facility- and census-tract-level characteristics of nursing facility by distance to nearest major roadway (A1 or A2) * and by high /low traffic density (length) of major roadways (A1 and A2) within 300 m buffer ^a^.

Characteristics	Distance to A1/A2 (m)		A1&A2 Traffic Density (Length) in 300 m Buffer (m)	
	≤150	>150	>0	=0
	*n* (%)	*n* (%)	*n* (%)	*n* (%)
All	3918 (28)	9925 (72)	6034 (41)	8746 (59)
Facility-level characteristics				
Ownership	3918 (28)	9925 (72)	6034 (41)	8746 (59)
For profit	2870 (29)	7160 (71)	4408 (41)	6255 (59)
Government	239 (30)	552 (70)	351 (39)	546 (61)
Non profit	809 (27)	2213 (73)	1275 (40)	1945 (60)
Certification	3918 (28)	9925 (72)	6034 (41)	8746 (59)
Medicaid	67 (25)	201 (75)	106 (35)	196 (65)
Medicare	128 (24)	411 (76)	205 (36)	372 (64)
Both	3723 (28)	9313 (72)	5723 (41)	8178 (59)
Council type	3918 (28)	9925 (72)	6034 (41)	8746 (59)
Family	8 (30)	19 (70)	13 (43)	17 (57)
Resident	2906 (28)	7314 (72)	4483 (41)	6398 (59)
Both	921 (28)	2311 (72)	1409 (40)	2073 (60)
None	83 (23)	281 (77)	129 (33)	258 (67)
Occupancy (beds)	3918 (28)	9925 (72)	6034 (41)	8746 (59)
>81	1826 (28)	4697 (72)	2922 (40)	4331 (60)
≤81	1877 (28)	4809 (72)	3112 (41)	4415 (59)
Tract-level characteristics				
% Urban	3706 (28)	9511 (72)	5728 (41)	8103 (59)
100%	1728 (24)	5525 (76)	2770 (37)	4714 (63)
<100%	1978 (33)	3986 (67)	2958 (47)	3389 (53)
% Non-White	3706 (28)	9511 (72)	5728 (41)	8103 (59)
>15%	1630 (25)	4940 (75)	2646 (38)	4227 (62)
≤15%	2076 (31)	4571 (69)	3082 (44)	3876 (56)
Household median income	3703 (28)	9506 (72)	5725 (41)	8096 (59)
>46,000	1902 (28)	4933 (72)	2848 (40)	4296 (60)
≤46,000	4801 (28)	4573 (72)	2877 (43)	3800 (57)

* The nursing homes with distance to major roadway equal to 0 were excluded for our study. ^a^ Data for Alaska, Hawaii, and Puerto Rico were excluded. All analyses was restricted to facility not in hospital.

**Table 2:** Prevalence ratios of facility- and census-tract-level characteristics associated with being located within 150 m of major roadways (A1 or A2) and exposure to high traffic density (length) within 300 m buffer of major roadways (A1 or A2) *.

Characteristics	Distance to A1 or A2 ≤150 m PR (95% CI)	High Traffic Density (Length) PR (95% CI)
Facility-level characteristics		
Ownership		
For profit	1.096 (1.001–1.201)	1.075 (0.992–1.165)
Government	1.184 (0.997–1.406)	0.981 (0.843–1.141)
Non profit	-	-
Certification		
Medicaid	0.834 (0.631–1.102)	0.773 (0.609–0.981)
Medicare	0.779 (0.637–0.953)	0.787 (0.662–0.937)
Both	-	-
Council type		
Family	1.425 (0.602–3.374)	1.529 (0.721–3.246)
Resident	1.345 (1.049–1.725)	1.401 (1.131–1.737)
Both	1.349 (1.044–1.744)	1.359 (1.089–1.697)
None	-	-
Occupancy (beds)		
>81	0.974 (0.904–1.049)	0.957 (0.896–1.022)
≤81	-	-
Tract-level characteristics		
% Urban		
100%	0.630 (0.584–0.680)	0.673 (0.629–0.721)
<100%	-	-
% Non-White		
>15%	0.727 (0.673–0.784)	0.787 (0.736–0.842)
≤15%	-	-
Household median income		
>46,000	0.979 (0.907–1.056)	0.876 (0.818–0.937)
≤46,000	-	-

* The nursing homes with distance to major roadway equal to 0 were excluded for our study. All analyses was restricted to facility not in hospital. Data for Alaska, Hawaii, and Puerto Rico were excluded.

**Table 3:** Prevalence ratios of facility- and census-tract-level characteristics associated with high/low traffic density (total AADT), and high/low traffic density (combo truck AADT) of major roadways within 300 m buffer *.

Characteristics	A1&A2 Total AADT in 300 m Buffer (Count/Year) PR (95% CI)	A1&A2 Combo Truck AADT in 300 m Buffer (Count/Year) PR (95% CI)
Facility-level characteristics		
Ownership		
For profit	1.373 (1.211–1.556)	1.500 (1.323–1.701)
Government	0.680 (0.532–0.868)	1.542 (1.216–1.955)
Non profit	-	-
Certification		
Medicaid	0.564 (0.378–0.842)	1.181 (0.803–1.737)
Medicare	2.034 (1.514–2.732)	0.844 (0.639–1.117)
Both	-	-
Council type		
Family	0.316 (0.083–1.198)	0.871 (0.277–2.733)
Resident	1.028 (0.724–1.459)	1.070 (0.754–1.519)
Both	1.149 (0.801–1.648)	0.939 (0.655–1.347)
None	-	-
Occupancy (beds)		
>81	2.444 (2.204–2.710)	1.025 (0.927–1.134)
≤81	-	-
Tract-level characteristics		
% Urban		
100%	4.426 (3.961–4.945)	0.893 (0.805–0.991)
<100%	-	-
% Non-White		
>15%	2.020 (1.818–2.245)	1.206 (1.087–1.338)
≤15%	-	-
Household median income		
>46,000	1.879 (1.692–2.086)	0.859 (0.775–0.953)
≤46,000	-	-

* The nursing homes with distance to major roadway equal to 0 were excluded for our study. All analyses were restricted to facility not in hospital. Data for Alaska, Hawaii, and Puerto Rico were excluded.

## Supplementary Materials

The following are available online at http://www.mdpi.com/1660-4601/15/3/487/s1, Table S1–S10: Supplementary Tables and Lists of Counties within Top 1%, 5% and 10%,
