# Supplementary material for: A National County-Level Assessment of U.S. Nursing Facility Characteristics Associated with Long-Term Exposure to Traffic Pollution in Older Adults
Source: Int J Environ Res Public Health. 2018 Mar 10;15(3):487. doi: 10.3390/ijerph15030487 (PMC5877032; doi:10.3390/ijerph15030487)
Supplement: Supplementary file 1 [file ijerph-15-00487-s001.pdf]

Details of facility-level care quality measures

Methods

The care-quality measures included overall quality rating (5 highest, 1 lowest) derived from rating of three domains, including individual quality rating for health inspection, individual quality rating for nursing home staffing levels (registered nurse +licensed practical nurse +nurse aide), and individual rating for quality measures (function and health status indicators based on Minimum Data Set (MDS) and Medicare claims data) (CMS 2017). Additionally, a stand-alone quality rating for registered nurse staffing levels was also included in the Nursing Home Compare datasets and thus was included in this analysis. Briefly, individual quality ratings for health inspection are based on the number, scope, and severity of deficiencies identified during the three most recent annual inspection surveys, as well as substantiated findings from the most recent 36 months of investigations about complaints. Individual ratings for staffing levels are based on registered nurse (RN) hours per resident day, and total staffing hours (RN+ licensed practical nurse (LPN) + nurse aide hours) per resident day. Other types of nursing-home staff, such as clerical or housekeeping staff, are not included in these staffing numbers. Individual ratings for quality measures are based on performance on 16 of the 24 MDS and Medicare claims-based quality measures, and that are based on MDS 3.0 assessments as well as hospital and emergency department claims (CMS 2017). These include nine long-stay measures and seven short-stay measures.

References

CMS. Design for Nursing Home Compare Five-Star Quality Rating System: Technical User's Guide, January 2017. Centers for Medicare and Medicaid Services, 2017.

Supplemental Tables ---4

Table 1. Facility- and census-tract-level characteristics for high/low traffic density (total AADT), and by high/low traffic density (combo truck AADT) of major roadways within 300 m buffer.*-----4

Table 2. Facility-level quality of care measures by distance to nearest major roadway (A1 or A2) * and by high /low traffic density (length) of major roadways (A1 and A2) within 300m buffer. [a] -----7

Table 3. Prevalence ratios of facility-level quality of care measures associated with being located within 150m of major roadways (A1 or A2) and exposure to high traffic density (length) within 300m buffer of major roadways (A1 or A2).* ---9

Table 4. Prevalence ratios of facility-level quality of care measures associated with exposure to high traffic density (total AADT) and high traffic density (combo truck AADT) within 300m buffer of major roadways*--- 11

Table 5. Prevalence ratios of facility- and census-tract-level characteristics* associated with being located within 150m of major roadways (A1 or A2)*, stratified by US region.------------------------- 13

Table 6. Prevalence ratios of facility- and census-tract-level characteristics associated with exposure to high traffic density (length) within 300m buffer of major roadways (A1 or A2)*, stratified by US region. -- 15

Table 7. Prevalence ratios of facility-level quality of care measures associated with being located within 150m of major roadways (A1 or A2)*, stratified by US region.----------------------------------- 17

Table 8. Prevalence ratios of facility-level quality of care measures associated with exposure to high traffic density (length) within 300m buffer of major roadways (A1 or A2), stratified by US region.* --- 19

Table 9: List of counties with highest percent (top 1%, 5%, 10%) of nursing residents near major roadways normalized to county area and total road length within the county ------------------------- 21

Table 10: List of counties with highest percent (Top 1%, 5%, 10%) of nursing facilities near major roadways normalized to county area and total road length within the county ------------------------- 29

Supplemental Tables

Table 1. Facility- and census-tract-level characteristics for high/low traffic density (total AADT), and by high/low traffic density (combo truck AADT) of major roadways within 300 m buffer.*

Characteristics	A1&A2 overall AADT (count/year)		A1&A2 Combo Truck AADT (count/year)	
	High<br>N (%)	Low<br>N (%)	High<br>N (%)	Low<br>N (%)
All	3023 (50.10)	3011 (49.90)	3047 (50.50)	2987 (49.50)
**Facility-level characteristics**				
**Ownership**	3023 (50.10)	3011 (49.90)	3047 (50.50)	2987 (49.50)
For profit	2326 (52.77)	2082 (47.23)	2318 (52.59)	2090 (47.41)
Government	125 (35.61)	226 (64.39)	187 (53.28)	164 (46.72)
Non profit	572 (44.86)	703 (55.14)	542 (42.51)	733 (57.49)
**Certification**	3023 (50.10)	3011 (49.90)	3047 (50.50)	2987 (49.50)
Medicaid	38 (35.85)	68 (64.15)	58 (54.72)	48 (45.28)
Medicare	137 (66.83)	68 (33.17)	95(46.34)	110 (53.66)
Both	2848 (49.76)	2875 (50.24)	2894 (50.57)	2829 (49.43)
**Council type**	3023 (50.10)	3011 (49.90)	3047 (50.50)	2987 (49.50)
Family	3 (23.08)	10 (76.92)	6 (49.15)	7 (53.85)
Resident	2220 (49.52)	2263 (50.48)	2300 (51.30)	2183 (48.70)
Both	737 (52.31)	672 (47.69)	677 (48.05)	732 (51.95)
None	63 (48.84)	66 (51.16)	64 (49.61)	65 (50.39)
**Occupancy (beds)**	3023 (50.10)	3011 (49.90)	3008 (49.85)	3026 (50.15)
>81	1789 (61.23)	1133 (38.77)	1458 (49.90)	1464 (50.10)
≤81	1234 (39.65)	1878 (60.35)	1550 (49.81)	1562 (50.19)
**Tract-level characteristics**				
**Regions**	3022 (50.09)	3011 (49.91)	3047 (50.51)	2986 (49.49)
Northeast	635 (52.22)	581 (47.78)	317 (26.07)	899 (73.93)
West	340 (54.93)	279 (45.07)	284 (45.88)	335 (54.12)
South	1273 (54.82)	1049 (45.18)	284 (45.88)	877 (37.77)
Midwest	774 (41.26)	1102 (58.74)	1001 (53.36)	875 (46.64)

**% Urban**	2869 (50.09)	2859 (49.91)	2869 (50.09)	2859 (49.91)
100%	1896 (68.45)	874 (31.55)	1347 (48.63)	1423 (51.37)
<100%	973 (32.89)	1985 (67.11)	1522 (51.45)	1436 (48.55)
**% Non-White**	2869 (50.09)	2859 (49.91)	2869 (50.09)	2859 (49.91)
>15%	1573 (59.45)	1073 (40.55)	1392 (42.61)	1254 (47.39)
≤15%	1296 (42.05)	1786 (57.95)	1477 (47.92)	1605 (52.08)
**Household median income**	2869 (50.11)	2856 (49.89)	2869 (50.11)	2856 (49.89)
>46,000	1590 (58.48)	1129 (41.52)	1306 (48.03)	1412 (51.97)
≤46,000	1279 (42.55)	1727 (57.45)	1563 (52.00)	1443 (48.00)
**Quality of care**				
**Overall rating**	2958 (50.02)	2956 (49.98)	2984 (50.46)	2930 (49.54)
5 (highest)	771 (51.06)	739 (48.94)	717 (47.48)	793 (52.52)
4	730 (50.31)	721 (49.69)	750 (51.69)	701 (48.31)
3	546 (47.64)	600 (52.36)	581 (50.70)	565 (49.30)
2	612 (51.43)	578 (48.57)	592 (49.75)	598 (50.25)
1	299 (48.46)	318 (51.54)	344 (55.75)	273 (44.25)
**Health Inspection Rating**	2958 (50.02)	2956 (49.98)	2984 (50.46)	2930 (49.54)
5 (highest)	297 (47.52)	328 (52.48)	309 (49.44)	316 (50.56)
4	642 (48.49)	682 (51.51)	653 (49.32)	671 (50.68)
3	688 (49.00)	716 (51.00)	713 (50.78)	691 (49.22)
2	736 (52.05)	678 (47.95)	719 (50.85)	695 (49.15)
1	595 (51.87)	522 (48.13)	590 (51.44)	557 (48.56)
**Quality rating**	2952 (49.97)	2955 (50.03)	2980 (50.45)	2927 (49.55)
5 (highest)	1281 (55.48)	1028 (44.52)	1156 (50.06)	1153 (49.94)
4	1063 (49.28)	1094 (50.72)	1075 (49.84)	1082 (50.16)
3	398 (44.37)	499 (55.63)	468 (52.17)	429 (47.83)
2	162 (39.90)	244 (60.10)	216 (53.20)	190 (46.80)
1	48 (34.78)	90 (65.22)	65 (47.10)	73 (52.90)
**Staffing rating**	2907 (49.87)	2922 (50.13)	2931 (50.28)	2898 (49.72)

5 (highest)	257 (51.61)	241 (48.39)	230 (46.18)	268 (53.82)
4	1244 (49.40)	1274 (50.60)	1232 (48.93)	1286 (51.07)
3	582 (49.40)	594 (50.51)	581 (49.40)	595 (50.60)
2	453 (50.11)	451 (49.89)	464 (51.33)	440 (48.67)
1	371 (50.61)	362 (49.39)	424 (57.84)	309 (42.16)
**RN [a] staffing rating**	2907 (49.87)	2922 (50.13)	2931 (50.28)	2898 (49.72)
5 (highest)	510 (46.75)	581 (53.25)	470 (43.08)	621 (56.92)
4	750 (50.37)	739 (49.63)	687 (46.14)	802 (53.86)
3	813 (49.97)	813 (50.03)	840 (51.69)	785 (48.31)
2	516 (49.95)	517 (50.05)	575 (55.66)	458 (44.34)
1	319 (53.98)	272 (46.02)	359 (60.74)	232 (39.26)

* Data for Alaska, Hawaii, and Puerto Rico were excluded. All analyses was restricted to facility not in hospital.

[a] RN: Registered Nurse

Table 2. Facility-level quality of care measures by distance to nearest major roadway (A1 or A2) * and by high /low traffic density (length) of major roadways (A1 and A2) within 300m buffer. [a]

	Distance to A1/A2 (m)		A1&A2 traffic density (length) in 300m buffer (m)	
	≤150	>150	>0	=0
**Quality of care**	**N (%)**	**N (%)**	**N (%)**	**N (%)**
**Overall rating**	3838 (28)	9654 (72)	5914 (41)	8448 (59)
5 (highest)	964 (28)	2517 (72)	1510 (41)	2195 (59)
4	984 (29)	2439 (71)	1451 (40)	2214 (60)
3	731 (30)	1715 (70)	1146 (44)	1471 (56)
2	759 (27)	2057 (73)	1190 (40)	1789 (60)
1	400 (30)	926 (70)	617 (44)	779 (56)
**Health Inspection Rating**	3838 (28)	9654 (72)	5914 (41)	8448 (59)
5 (highest)	399 (29)	979 (71)	625 (42)	850 (58)
4	877 (28)	2208 (72)	1324 (40)	1972 (60)
3	906 (29)	2232 (71)	1404 (42)	1963 (58)
2	924 (29)	2251 (71)	1414 (42)	1961 (58)
1	732 (27)	1984 (73)	1147 (40)	1702 (60)
**Quality rating**	38834 (28)	9646 (72)	5907 (41)	8442 (59)
5 (highest)	1498 (27)	4084 (73)	2309 (39)	3607 (61)
4	1405 (29)	3461 (71)	2157 (42)	3002 (58)
3	579 (30)	1359 (70)	897 (43)	1193 (57)
2	267 (33)	551 (67)	406 (46)	470 (54)
1	85 (31)	191 (69)	138 (45)	170 (55)
**Staffing rating**	3784 (28)	9498 (72)	5829 (41)	8309 (59)
5 (highest)	307 (25)	922 (75)	498 (38)	817 (62)
4	1622 (28)	4200 (72)	2518 (41)	3647 (59)
3	782 (30)	1867 (70)	1176 (42)	1652 (58)
2	609 (31)	1369 (69)	904 (43)	1219 (57)
1	464 (29)	1140 (71)	773 (43)	974 (57)
**RN [b] staffing rating**	3784 (28)	9498 (72)	5829 (41)	8309 (59)
5 (highest)	688 (27)	1872 (73)	1091 (40)	1612 (60)
4	992 (28)	2525 (72)	1489 (40)	2230 (60)

3	1080 (30)	2544 (70)	1625 (42)	2249 (58)
2	654 (30)	1543 (70)	1033 (44)	1337 (56)
1	370 (27)	1014 (73)	591 (40)	881 (60)

* The nursing homes with distance to major roadway equal to 0 were excluded for our study.

[a] Data for Alaska, Hawaii, and Puerto Rico were excluded. All analyses was restricted to facility not in hospital.

[b] RN: Registered Nurses

Table 3. Prevalence ratios of facility-level quality of care measures associated with being located within 150m of major roadways (A1 or A2) and exposure to high traffic density (length) within 300m buffer of major roadways (A1 or A2).*

Quality of care	Distance to A1 or A2 ≤150 m PR (95% CI)	High traffic density (length) PR (95% CI)
**Overall rating**		
5 (highest)	0.887 (0.772 – 1.019)	**0.869 (0.767 – 0.984)**
4	0.934 (0.813 – 1.073)	**0.827 (0.730 – 0.937)**
3	0.987 (0.853 – 1.142)	0.984 (0.863 – 1.121)
2	**0.854 (0.740 – 0.986)**	**0.840 (0.7398 – 0.955)**
1	-	-
**Health Inspection Rating**		
5 (highest)	1.105 (0.956 – 1.276)	1.070 (0.967 – 1.184)
4	1.077 (0.959 – 1.208)	1.061 (0.959 – 1.175)
3	1.100 (0.981 – 1.234)	0.996 (0.899 – 1.104)
2	1.113 (0.956 – 1.276)	1.070 (0.960 – 1.240)
1	-	-
**Quality rating**		
5 (highest)	0.824 (0.634 – 1.071)	**0.789 (0.626 – 0.993)**
4	0.712 (0.701 – 1.187)	0.885 (0.702 – 1.115)
3	0.957 (0.728 – 1.258)	0.926 (0.728 – 1.178)
2	1.089 (0.811 – 1.462)	1.064 (0.820 – 1.381)
1	-	-
**Staffing rating**		
5 (highest)	**0.818 (0.691 – 0.968)**	**0.810 (0.699 – 0.938)**
4	0.949 (0.840 – 1.072)	0.917 (0.823 – 1.023)
3	1.029 (0.898 – 1.180)	0.946 (0.838 – 1.068)
2	1.093 (0.946 – 1.262)	0.985 (0.866 – 1.121)
1	-	-
**RN [a] staffing rating**		
5 (highest)	1.007 (0.869 – 1.167)	1.009 (0.886 – 1.148)
4	1.077 (0.936 – 1.238)	0.995 (0.880 – 1.126)
3	**1.163 (1.013 – 1.238)**	1.007 (0.953 – 1.217)

2	1.162 (0.869 – 1.167)	**1.152 (1.009 – 1.314)**
1	-	-

*The nursing homes with distance to major roadway equal to 0 were excluded for our study. All analyses was restricted to facility not in hospital. Data for Alaska, Hawaii, and Puerto Rico were excluded.

[a] RN: Registered Nurses

Table 4. Prevalence ratios of facility-level quality of care measures associated with exposure to high traffic density (total AADT) and high traffic density (combo truck AADT) within 300m buffer of major roadways*

Characteristics	A1&A2 total AADT in 300m buffer (count/year) PR (95% CI)	A1&A2 combo truck AADT in 300m buffer (count/year) PR (95% CI)
**Quality of care**		
**Overall rating**		
5 (highest)	1.110 (0.920 – 1.338)	**0.718 (0.594 – 0.866)**
4	1.077 (0.892 – 1.300)	0.849 (0.703 – 1.026)
3	0.968 (0.796 – 1.177)	**0.816 (0.670 – 0.993)**
2	1.126 (0.927 – 1.368)	**0.786 (0.646 – 0.995)**
1	-	-
**Health Inspection Rating**		
5 (highest)	0.840 (0.691 – 1.021)	0.923 (0.760 – 1.122)
4	0.873 (0.746 – 1.023)	0.919 (0.784 – 1.076)
3	0.891 (0.763 – 1.042)	0.974 (0.833 – 1.139)
2	1.007 (0.862 – 1.177)	0.977 (0.836 – 1.151)
1	-	-
**Quality rating**		
5 (highest)	**2.336 (1.630 – 3.348)**	1.126 (0.798 – 1.588)
4	**1.822 (1.271 – 2.612)**	1.116 (0.790 – 1.575)
3	**1.495 (1.029 – 2.174)**	1.225 (0.855 – 1.754)
2	1.245 (0.832 – 1.862)	1.276 (0.867 – 1.879)
1	-	-
**Staffing rating**		
5 (highest)	1.040 (0.829 – 1.307)	**0.625 (0.497 – 0.787)**
4	0.953 (0.808 – 1.123)	**0.698 (0.591 – 0.824)**
3	0.956 (0.795 – 1.150)	**0.712 (0.591 – 0.857)**
2	0.980 (0.807 – 1.191)	**0.769 (0.632 – 0.935)**
1	-	-
**RN [a] staffing rating**		
5 (highest)	**0.749 (0.613 – 0.915)**	**0.489 (0.399 – 0.600)**

4	0.866 (0.715 – 1.048)	**0.554 (0.456 – 0.672)**
3	0.852 (0.705 – 1.029)	**0.692 (0.571 – 0.838)**
2	**0.851 (0.695 – 0.915)**	**0.811 (0.660 – 0.997)**
1	**-**	**-**

*The nursing homes with distance to major roadway equal to 0 were excluded for our study. All analyses was restricted to facility not in hospital. Data for Alaska, Hawaii, and Puerto Rico were excluded.

[a] RN: Registered Nurses

Table 5. Prevalence ratios of facility- and census-tract-level characteristics* associated with being located within 150m of major roadways (A1 or A2)*, stratified by US region.

	Logistic Regression (Distance to A1/A2 ≤150m)			
**Characteristics**	**West PR (95% CI)**	**Midwest PR (95% CI)**	**South PR (95% CI)**	**Northeast PR (95% CI)**
**Facility-level characteristics**				
**Ownership**				
For profit	0.988 (0.697 – 1.402)	**1.166 (1.002 – 1.356)**	**1.194 (1.005 – 1.420)**	1.169 (0.971 – 1.408)
Government	1.794 (0.908 – 3.547)	**1.286 (1.010 – 1.637)**	1.243 (0.899 – 1.719)	0.870 (0.520 – 1.456)
Non profit	-	-	-	-
**Certification**				
Medicaid	1.508 (0.843 – 2.698)	0.674 (0.416 – 1.093)	0.923 (0.557 – 1.530)	1.783 (0.705 – 4.509)
Medicare	1.073 (0.676 – 1.704)	0.911 (0.576 – 1.440)	**0.653 (0.468 – 0.912)**	0.964 (0.626 – 1.486)
Both	-	-	-	-
**Council type**				
Family	0.947 (0.103 – 8.699)	1.323 (0.142 – 12.356)	1.060 (0.291 – 3.855)	1.579 (0.205 – 12.173)
Resident	0.795 (0.448 – 1.412)	**2.029 (1.304 – 3.158)**	1.020 (0.631 – 1.650)	0.891 (0.497 – 1.598)
Both	0.944 (0.507 – 1.756)	**1.793 (1.130 – 2.844)**	0.923 (0.565 – 1.507)	0.871 (0.480 – 1.580)
None	-	-	-	-
**Occupancy (beds)**				
>81	**0.772 (0.646 – 0.924)**	0.902 (0.786 – 1.035)	0.916 (0.809 – 1.036)	**0.778 (0.609 – 0.993)**
≤81	-	-	-	-
**Tract-level characteristics**				
**% Urban**				
100%	**0.581 (0.441 – 0.765)**	**0.737 (0.643 – 0.844)**	**0.614 (0.540 – 0.698)**	**0.649 (0.543 – 0.776)**
<100%	-	-	-	-
**% Non-White**				
>15%	**0.704 (0.544 – 0.911)**	**0.742 (0.635 – 0.867)**	**0.812 (0.710 – 0.928)**	**0.621 (0.518 – 0.745)**
≤15%	-	-	-	-
**Household median income**				

>46,000	0.955 (0.747 – 1.220)	1.061 (0.929 – 1.213)	**0.829 (0.728 – 0.945)**	1.160 (0.957 – 1.407)
≤46,000	-	-	-	-

*The nursing homes with distance to major roadway equal to 0 were excluded for our study. Data for Alaska, Hawaii, and Puerto Rico were excluded. All analyses was restricted to facility not in hospital.

[a] RN: Registered Nurses

Table 6. Prevalence ratios of facility- and census-tract-level characteristics associated with exposure to high traffic density (length) within 300m buffer of major roadways (A1 or A2)*, stratified by US region.

	Logistic Regression (High traffic density (length))			
**Characteristics**	**West PR (95% CI)**	**Midwest PR (95% CI)**	**South PR (95% CI)**	**Northeast PR (95% CI)**
**Facility-level characteristics**				
**Ownership**				
For profit	0.954 (0.729 – 1.250)	1.026 (0.899 – 1.171)	**1.197 (1.027 – 1.394)**	**1.213 (1.022 – 1.439)**
Government	1.089 (0.615 – 1.929)	1.024 (0.827 – 1.268)	1.025 (0.770 – 1.366)	0.851 (0.547 – 1.324)
Non profit	-	-	-	-
**Certification**				
Medicaid	0.967 (0.579 – 1.614)	0.867 (0.588 – 1.280)	0.730 (0.477 – 1.116)	1.090 (0.452 – 2.629)
Medicare	1.046 (0.729 – 1.500)	0.899 (0.599 – 1.351)	**0.677 (0.510 – 0.899)**	0.818 (0.551 – 1.214)
Both	-	-	-	-
**Council type**				
Family	0.889 (0.162 – 4.873)	1.736 (0.280 – 10.751)	1.412 (0.438 – 4.552)	1.800 (0.278 – 11.635)
Resident	0.868 (0.545 – 1.384)	**1.692 (1.183 – 2.419)**	1.392 (0.905 – 2.142)	1.126 (0.657 – 1.931)
Both	0.904 (0.545 – 1.499)	1.434 (0.985 – 2.089)	1.291 (0.832 – 2.002)	1.021 (0.589 – 1.769)
None	-	-	-	-
**Occupancy (beds)**				
>81	**0.748 (0.632 – 0.885)**	**0.856 (0.758 – 0.967)**	0.946 (0.847 – 1.057)	**0.818 (0.677 – 0.988)**
≤81	-	-	-	-
**Tract-level characteristics**				
**% Urban**				
100%	**0.536 (0.430 – 0.668)**	**0.744 (0.660 – 0.840)**	**0.677 (0.603 – 0.759)**	**0.724 (0.610 – 0.858)**
<100%	-	-	-	-
**% Non-White**				
>15%	**0.700 (0.570 – 0.859)**	**0.708 (0.618 – 0.812)**	**0.852 (0.754 – 0.963)**	**0.755 (0.640 – 0.892)**
≤15%	-	-	-	-
**Household median income**				

>46,000	**0.910 (0.752 – 1.103)**	0.917 (0.814 – 1.032)	**0.782 (0.696 – 0.879)**	0.969 (0.810 – 1.159)
≤46,000	-	-	-	-

*The nursing homes with distance to major roadway equal to 0 were excluded for our study. Data for Alaska, Hawaii, and Puerto Rico were excluded. All analyses were restricted to facility not in hospital.

[a] RN: Registered Nurses

Table 7. Prevalence ratios of facility-level quality of care measures associated with being located within 150m of major roadways (A1 or A2)*, stratified by US region.

	Logistic Regression (Distance to A1/A2 ≤150m)			
**Quality of care**	**West PR (95% CI)**	**Midwest PR (95% CI)**	**South PR (95% CI)**	**Northeast PR (95% CI)**
**Overall rating**				
5 (highest)	1.347 (0.742 – 2.443)	0.822 (0.643 – 1.052)	1.088 (0.874 – 1.156)	0.862 (0.622 – 1.196)
4	1.373 (0.750 – 2.514)	0.841 (0.660 – 1.072)	1.071 (0.863 – 1.328)	0.983 (0.705 – 1.371)
3	1.085 (0.572 – 2.058)	0.853 (0.661 – 1.102)	1.189 (0.950 – 1.489)	1.104 (0.777 – 1.569)
2	1.374 (0.738 – 2.555)	**0.731 (0.566 – 0.944)**	0.927 (0.743 – 1.156)	0.935 (0.664 – 1.315)
1	-	-	-	-
**Health Inspection Rating**				
5 (highest)	1.361 (0.874 – 2.117)	1.037 (0.801 – 1.343)	1.207 (0.950 – 1.533)	0.919 (0.666 – 1.233)
4	1.012 (0.696 – 1.473)	1.178 (0.962 – 1.443)	1.164 (0.960 – 1.410)	0.816 (0.631 – 1.055)
3	1.088 (0.754 – 1.570)	1.116 (0.911 – 1.367)	1.152 (0.952 – 1.394)	1.015 (0.785 – 1.313)
2	0.983 (0.676 – 1.430)	1.142 (0.934 – 1.397)	1.138 (0.940 – 1.376)	1.087 (0.842 – 1.403)
1	-	-	-	-
**Quality rating**				
5 (highest)	0.786 (0.321 – 1.927)	0.830 (0.541 – 1.273)	0.774 (0.522 – 1.146)	1.464 (0.528 – 4.061)
4	0.949 (0.385 – 2.341)	0.862 (0.562 – 1.322)	0.760 (0.512 – 1.128)	1.861 (0.671 – 5.166)
3	0.763 (0.296 – 1.967)	0.868 (0.556 – 1.355)	0.929 (0.616 – 1.401)	2.019 (0.712 – 5.725)
2	1.014 (0.365 – 2.821)	1.100 (0.682 – 1.773)	0.952 (0.611 – 1.484)	2.076 (0.693 – 6.221)
1	-	-	-	-
**Staffing rating**				
5 (highest)	1.225 (0.547 – 2.742)	0.741 (0.547 – 1.003)	0.835 (0.599 – 1.163)	**1.499 (1.020 – 2.202)**
4	1.065 (0.495 – 2.290)	0.846 (0.671 – 1.068)	1.148 (0.964 – 1.368)	**1.551 (1.132 – 2.123)**
3	1.046 (0.470 – 2.326)	0.941 (0.728 – 1.216)	1.167 (0.958 – 1.423)	**1.533 (1.088 – 2.161)**
2	1.074 (0.458 – 2.517)	0.978 (0.747 – 1.280)	1.127 (0.916 – 1.387)	**1.626 (1.138 – 2.323)**
1	-	-	-	-
**RN [a] staffing rating**				
5 (highest)	**2.182 (1.067 – 4.463)**	0.830 (0.612 – 1.125)	1.030 (0.780 – 1.359)	**1.878 (1.192 – 2.958)**
4	1.780 (0.867 – 3.651)	0.835 (0.620 – 1.123)	**1.319 (1.076 – 1.617)**	**1.883 (1.202 – 2.949)**

3	1.738 (0.839 – 3.601)	0.845 (0.627 – 1.138)	**1.361 (1.133 – 1.635)**	**2.060 (1.306 – 3.248)**
2	1.618 (0.728 – 3.600)	1.075 (0.779 – 1.482)	1.145 (0.945 – 1.386)	1.534 (0.940 – 2.505)
1	-	-	-	-

*The nursing homes with distance to major roadway equal to 0 were excluded for our study. Data for Alaska, Hawaii, and Puerto Rico were excluded. All analyses was restricted to facility not in hospital.

[a] RN: Registered Nurses

Table 8. Prevalence ratios of facility-level quality of care measures associated with exposure to high traffic density (length) within 300m buffer of major roadways (A1 or A2), stratified by US region.*

	Logistic Regression (High traffic density (length))			
**Quality of care**	**West PR (95% CI)**	**Midwest PR (95% CI)**	**South PR (95% CI)**	**Northeast PR (95% CI)**
**Overall rating**				
5 (highest)	0.954 (0.620 – 1.468)	0.912 (0.729 – 1.140)	1.007 (0.828 – 1.226)	0.808 (0.595 – 1.097)
4	0.923 (0.594 – 1.435)	**0.794 (0.636 – 0.991)**	0.921 (0.759 – 1.117)	0.858 (0.628 – 1.172)
3	0.979 (0.618 – 1.549)	0.904 (0.716 – 1.140)	1.139 (0.930 – 1.394)	1.083 (0.778 – 1.507)
2	1.124 (0.716 – 1.764)	**0.744 (0.590 – 0.938)**	0.920 (0.756 – 1.120)	0.830 (0.603 – 1.143)
1	-	-	-	-
**Health Inspection Rating**				
5 (highest)	0.982 (0.710 – 1.251)	**1.255 (1.002 – 1.367)**	1.078 (0.870 – 1.337)	0.932 (0.693 – 1.254)
4	0.865 (0.649 – 1.154)	1.111 (0.926 – 1.332)	1.046 (0.881 – 1.242)	0.813 (0.641 – 1.031)
3	0.872 (0.656 – 1.158)	1.138 (0.951 – 1.362)	1.094 (0.923 – 1.296)	1.035 (0.814 – 1.316)
2	0.943 (0.710 – 1.251)	1.142 (0.955 – 1.367)	1.096 (0.926 – 1.299)	1.003 (0.790 – 1.275)
1	-	-	-	-
**Quality rating**				
5 (highest)	**0.375 (0.191 – 0.739)**	0.791 (0.542 – 1.154)	0.938 (0.660 – 1.333)	1.128 (0.479 – 2.660)
4	**0.453 (0.229 – 0.896)**	0.795 (0.545 – 1.161)	1.009 (0.709 – 1.435)	1.424 (0.603 – 3.362)
3	**0.388 (0.190 – 0.792)**	0.851 (0.575 – 1.262)	1.116 (0.772 – 1.613)	1.620 (0.671 – 3.911)
2	0.721 (0.329 – 1.541)	0.991 (0.648 – 1.516)	1.092 (0.732 – 1.628)	2.053 (0.797 – 5.285)
1	-	-	-	-
**Staffing rating**				
5 (highest)	1.059 (0.572 – 1.959)	**0.763 (0.583 – 0.998)**	0.972 (0.730 – 1.295)	1.199 (0.849 – 1.693)
4	0.956 (0.535 – 1.711)	0.918 (0.743 – 1.134)	1.038 (0.888 – 1.213)	**1.325 (1.002 – 1.751)**
3	0.833 (0.452 – 1.535)	0.950 (0.752 – 1.199)	1.060 (0.888 – 1.266)	1.269 (0.934 – 1.724)
2	0.876 (0.456 – 1.682)	0.981 (0.768 – 1.253)	1.033 (0.859 – 1.243)	1.328 (0.899 – 1.706)
1	-	-	-	-
**RN [a] staffing rating**				
5 (highest)	**1.723 (1.054 – 2.818)**	1.002 (0.759 – 1.322)	0.980 (0.767 – 1.253)	**1.783 (1.201 – 2.649)**
4	1.330 (0.812 – 2.180)	0.969 (0.739 – 1.271)	1.153 (0.961 – 1.383)	**1.595 (1.080 – 2.356)**

3	1.345 (0.814 – 2.221)	0.997 (0.760 – 1.309)	**1.196 (1.015 – 1.408)**	**1.700 (1.143 – 2.527)**
2	1.315 (0.750 – 2.304)	1.212 (0.902 – 1.628)	1.111 (0.939 – 1.315)	**1.552 (1.012 – 2.379)**
1	-	-	-	-

* Data for Alaska, Hawaii, and Puerto Rico were excluded. All analyses was restricted to facility not in hospital.

[a] RN: Registered Nurses

Table 9: List of counties with highest percent (top 1%, 5%, 10%) of nursing residents near major roadways normalized to county area and total road length within the county

Percentile	State	County Name
	VA	FAIRFAX CITY
	VA	WINCHESTER
	VA	NORTON
	VA	HOPEWELL
	VA	EMPORIA
	NY	NEW YORK
	VA	WAYNESBORO
	VA	HARRISONBURG
	VA	STAUNTON
	RI	BRISTOL
	VA	ALEXANDRIA
	VA	PETERSBURG
	NY	RICHMOND
	VA	ARLINGTON
	NY	BRONX
	NY	KINGS
Top 1%	VA	PORTSMOUTH
	CA	SAN FRANCISCO
	DC	DISTRICT OF COLUMBIA
	WV	HANCOCK
	VA	DANVILLE

NM	LOS ALAMOS
NJ	HUDSON
VA	ROANOKE CITY
VA	LYNCHBURG
VA	HAMPTON
KY	ROBERTSON
VA	RICHMOND CITY
MA	SUFFOLK
TN	MOORE
MO	ST. LOUIS CITY
IN	UNION
IN	FAYETTE
TX	ROCKWALL
VA	NEWPORT NEWS
TN	TROUSDALE
IL	EDWARDS
RI	NEWPORT
IL	WABASH
PA	MONTOUR
WV	OHIO
NJ	UNION
VA	NORFOLK
GA	OCONEE

GA	CLAY
NJ	ESSEX
GA	STEPHENS
MD	BALTIMORE CITY
VA	WESTMORELAND
IN	FLOYD
TX	ARANSAS
KY	TRIMBLE
GA	BARROW
VA	WARREN
GA	PICKENS
VA	FLUVANNA
TN	CLAY
WV	JEFFERSON
KY	CARLISLE
WV	CALHOUN
TN	HANCOCK
IL	BROWN
GA	LANIER
IN	SCOTT
IA	JEFFERSON
IN	SWITZERLAND
NC	POLK

	CO	DENVER
	IA	HUMBOLDT
	GA	CHATTOOGA
	GA	HARALSON
	GA	BEN HILL
	GA	COOK
	TN	CANNON
	KY	NICHOLAS
	GA	CATOOSA
	GA	JOHNSON
	KY	SPENCER
	TX	RAINS
	NC	CURRITUCK
	KY	HANCOCK
	GA	CANDLER
Top 5%	GA	TREUTLEN
	VA	ISLE OF WIGHT
	MO	GENTRY
	SD	CLAY
	NC	NEW HANOVER
	IN	HOWARD
	IA	LOUISA
	KY	FRANKLIN

MI	LAKE
NE	COLFAX
WI	GREEN LAKE
KY	POWELL
WV	MARION
RI	KENT
KY	EDMONSON
TN	MACON
KY	ANDERSON
KY	CAMPBELL
KY	CLINTON
KY	MARTIN
NE	GOSPER
GA	UPSON
GA	LEE
VA	JAMES CITY
KY	BOYLE
MI	ARENAC
NC	LEE
GA	GILMER
GA	POLK
VA	LANCASTER
GA	PULASKI

OK	WASHINGTON
IA	MITCHELL
TN	RHEA
MO	RANDOLPH
IL	MOULTRIE
VA	ORANGE
NC	GREENE
NJ	PASSAIC
PA	CAMERON
IN	DECATUR
GA	WALTON
WI	PEPIN
KY	KENTON
MN	WASECA
IA	IDA
TN	CHEATHAM
WV	MARSHALL
MN	RAMSEY
MS	QUITMAN
GA	IRWIN
IA	APPANOOSE
KY	LYON
NE	WASHINGTON

NE	SARPY
GA	JONES
WV	CABELL
KY	BRACKEN
IN	STARKE
SD	MOODY
GA	MURRAY
IN	MARTIN
IA	ADAMS
IA	PALO ALTO
IA	MAHASKA
KS	WOODSON
GA	MORGAN
IL	CLAY
WV	WETZEL
MN	PIPESTONE
VA	ROANOKE
GA	CALHOUN
NE	STANTON
NY	YATES
NC	SCOTLAND
MI	EMMET
NY	SCHENECTADY

	IL	JERSEY
	MS	CLAY
	NC	HERTFORD
	KY	HARRISON
	IN	ADAMS
Top 10%	MO	RAY

Table 10: List of counties with highest percent (Top 1%, 5%, 10%) of nursing facilities near major roadways normalized to county area and total road length within the county

Percentile	State	County Name
	VA	FAIRFAX CITY
	VA	NORTON
	VA	WINCHESTER
	VA	EMPORIA
	VA	HOPEWELL
	VA	STAUNTON
	VA	WAYNESBORO
	RI	BRISTOL
	VA	HARRISONBURG
	VA	PETERSBURG
	VA	ALEXANDRIA
	NM	LOS ALAMOS
	VA	DANVILLE
	NY	NEW YORK
	TN	TROUSDALE
	TN	MOORE
Top 1%	IN	UNION
	VA	ROANOKE CITY
	VA	LYNCHBURG
	KY	ROBERTSON
	VA	ARLINGTON

NY	RICHMOND
WV	HANCOCK
VA	PORTSMOUTH
IL	EDWARDS
IL	WABASH
VA	RICHMOND CITY
NY	BRONX
GA	CLAY
TX	RAINS
GA	OCONEE
NY	KINGS
GA	STEPHENS
TN	HANCOCK
IL	BROWN
GA	BARROW
VA	WESTMORELAND
KY	TRIMBLE
IN	FAYETTE
KY	CARLISLE
VA	WARREN
VA	FLUVANNA
TN	CLAY
GA	LANIER

WV	OHIO
IN	SWITZERLAND
WV	CALHOUN
TX	ROCKWALL
IA	ADAMS
PA	MONTOUR
GA	COOK
NC	CURRITUCK
TN	CANNON
VA	HAMPTON
MO	ST. LOUIS CITY
TX	MORRIS
KY	NICHOLAS
GA	CHATTOOGA
NE	GOSPER
GA	CATOOSA
GA	CANDLER
KY	SPENCER
NJ	HUDSON
WI	GREEN LAKE
GA	JOHNSON
CA	SAN FRANCISCO
VA	LANCASTER

	KY	HANCOCK
	NC	GREENE
	WV	JEFFERSON
	VA	NEWPORT NEWS
	MI	LAKE
	KY	BOYLE
	GA	TREUTLEN
	KS	WOODSON
	IA	LUCAS
	KY	POWELL
	PA	CAMERON
	GA	LUMPKIN
	MS	STONE
	TN	MACON
	GA	WARREN
Top 5%	KY	EDMONSON
	KY	FRANKLIN
	WI	PEPIN
	IA	HUMBOLDT
	IA	WORTH
	GA	IRWIN
	IL	KENDALL
	KY	MARTIN

KY	ANDERSON
IN	STARKE
IN	OWEN
KY	CLINTON
GA	PULASKI
MS	QUITMAN
WV	WETZEL
VA	ORANGE
MS	SHARKEY
AR	LEE
GA	POLK
GA	GILMER
GA	WALTON
TN	DECATUR
GA	LEE
GA	MURRAY
VA	NOTTOWAY
KY	BRACKEN
IN	SCOTT
TN	CHEATHAM
SD	MOODY
IN	PIKE
TN	SCOTT

NC	HERTFORD
GA	CALHOUN
MI	EMMET
NE	STANTON
GA	NEWTON
MO	CLARK
GA	UPSON
TX	ARANSAS
IN	PARKE
ID	PAYETTE
MN	ISANTI
GA	BEN HILL
TN	JACKSON
KY	ROWAN
IN	STEUBEN
GA	MORGAN
MO	MORGAN
KY	FULTON
VA	NORFOLK
NE	BOYD
MO	CLINTON
MO	LEWIS
KY	MEADE

LA	ST. JOHN THE BAPTIST
IA	JEFFERSON
MO	PERRY
IA	GREENE
RI	KENT
MD	SOMERSET
NC	WARREN
TN	WHITE
WV	LEWIS
NE	VALLEY
IL	CLAY
VA	BUCHANAN
TN	SMITH
KY	CAMPBELL
VA	CULPEPER
VA	JAMES CITY
DC	DISTRICT OF COLUMBIA
GA	MARION
MO	DEKALB
KY	FLEMING
MI	MANISTEE
GA	GREENE
AR	CLAY

	GA	BRANTLEY
	LA	ST. JAMES
	NJ	UNION
	OK	MARSHALL
	KY	LARUE
Top 10%	IN	FLOYD
